# Shaping the Neurovascular Unit Exploiting Human Brain Organoids

**DOI:** 10.1007/s12035-024-03998-9

**Published:** 2024-02-09

**Authors:** Mafalda Rizzuti, Valentina Melzi, Lorenzo Brambilla, Lorenzo Quetti, Luca Sali, Linda Ottoboni, Megi Meneri, Antonia Ratti, Federico Verde, Nicola Ticozzi, Giacomo Pietro Comi, Stefania Corti, Elena Abati

**Affiliations:** 1 Neurology Unit, Foundation IRCCS Ca’ Granda Ospedale Maggiore Policlinico, Milan, Italy; 2 Dino Ferrari Centre, Department of Pathophysiology and Transplantation (DEPT), Università degli Studi di Milano, Milan, Italy; 3 Department of Neurology and Laboratory of Neuroscience, IRCCS Istituto Auxologico Italiano, Milan, Italy; 4 Department Medical Biotechnology and Translational Medicine, Università degli Studi di Milano, Milan, Italy; 5 Neuromuscular and Rare Diseases Unit, Department of Neuroscience, Foundation IRCCS Ca’ Granda Ospedale Maggiore Policlinico, Milan, Italy

**Keywords:** Pluripotent stem cells, Vascular system, Cerebral organoids, Vascular organoids, Circulation, Microcirculation

## Abstract

Brain organoids, three-dimensional cell structures derived from pluripotent stem cells, closely mimic key aspects of the human brain *in vitro*, providing a powerful tool for studying neurodevelopment and disease. The neuroectodermal induction protocol employed for brain organoid generation primarily gives rise to the neural cellular component but lacks the vital vascular system, which is crucial for the brain functions by regulating differentiation, migration, and circuit formation, as well as delivering oxygen and nutrients. Many neurological diseases are caused by dysfunctions of cerebral microcirculation, making vascularization of human brain organoids an important tool for pathogenetic and translational research. Experimentally, the creation of vascularized brain organoids has primarily focused on the fusion of vascular and brain organoids, on organoid transplantation *in vivo*, and on the use of microfluidic devices to replicate the intricate microenvironment of the human brain *in vitro*. This review summarizes these efforts and highlights the importance of studying the neurovascular unit in a forward-looking perspective of leveraging their use for understanding and treating neurological disorders.

**Graphical Abstract:**

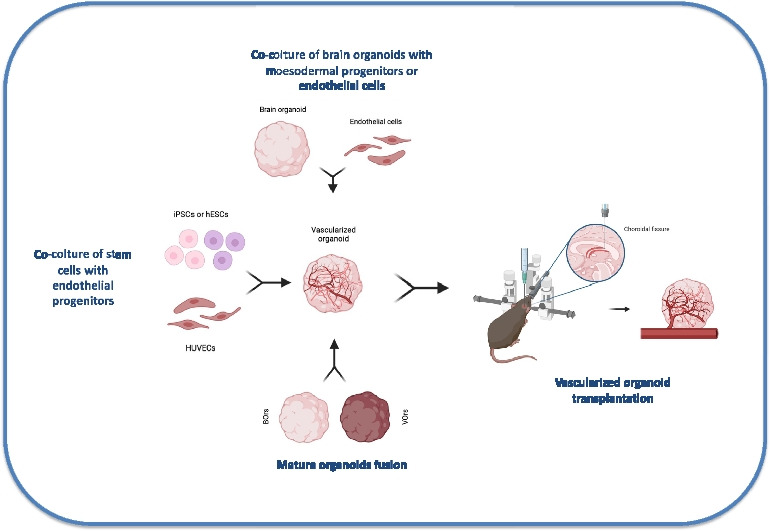

## Introduction

Organoids are complex three-dimensional (3D) cellular systems derived from pluripotent stem cells (PSCs) or adult stem cells [1, 2]. Concerning the nervous system’s organoids, when the originating cells are cultured in suspension under specific conditions that reproduce embryonic development, they form structures that mimic the organ architectures and functions, including the central nervous system (CNS) as a whole (whole-brain organoids) or specific CNS areas (regional organoids) [3]. As cells develop within brain organoids, they follow a developmental timeline that is similar to *in vivo* neurogenesis [3]. Brain organoids are able to generate spontaneous neural activity, form functional synapses, and support interneuron migration or axonal projection, as well as interact and fuse with adjacent organoids giving rise to assembloids, mimicking the architecture and complex interactions of CNS tissues [4, 5]. Additionally, transcriptomic and epigenetic studies confirmed that brain organoids recapitulate the key molecular features of the human embryonic/fetal brain [4].

The main source of cells used for brain organoid generation is induced pluripotent stem cells (iPSCs) [6] and embryonic stem cells (ESCs) [7], both widely employed. iPSCs are derived from somatic cells primarily of the skin or of the blood, which are reprogrammed into embryonic-like states by administration of specific pluripotency transcription factors. Unlike ESCs, iPSCs do not carry the ethical concerns associated with ESCs while fully reflecting the patient’s genetic background [6, 8, 9]. Notably, iPSCs are able to differentiate in several cell types, including neuronal cells, making them particularly suitable for studying neurodegenerative disorders.

To date, disease modeling has largely relied on two-dimensional (2D) cell cultures, human biopsy specimens, and animal models. Nevertheless, the majority of human tissues are difficult to biopsy, and their use is strictly regulated, while 2D cell cultures do not fully mimic the structural organization and functions of human tissues, poorly attempting to replicate the real *in vivo* conditions [8]. As regards *in vivo* animal models, despite their ability to replicate the complexity of living organisms better than *in vitro* ones, their utility is limited by interspecies differences.

Recently, iPSC-derived 3D models, grown in a way that cells interact with each other and their surroundings more naturally, mimicking the *in vivo* conditions to a greater extent, have become key tool for disease modeling and for the development of new therapeutic strategies, closely resembling the *in vivo* microenvironment. In this context, iPSC-derived organoids, free floating 3D models, offer the chance to deeply investigate physiological and pathological mechanisms in a specific genetic background. Particularly, brain organoids have attracted huge interest to study neural development and as innovative tools for drug discovery and regenerative medicine [10]. The exploitation of organoid potential can be further extended to the generation of multi-unit structure called assembloid, a more sophisticated *in vitro* model, which attempts to recapitulate intercellular interactions among different organ-like structures [11]. Assembloids are self-organizing cellular entities emerging from the integration of distinct organoids or derived from the combination with specialized cell populations [5, 12].

So far, iPSC-derived brain organoids have been generated to model a large range of both developmental and degenerative brain disorders [13–17]. Nonetheless, their major limitation, as a model, is the lack of a vascular system for transporting nutrients or drugs, which *in vivo* occurs through microvascular cells and structures. The absence of a neurovascular system limits organoid growth, neurogenesis, and functions, restricting their potential applications [18–22].

Because of that, vascularization of brain organoids is one of the main sought-after advancements in the field, allowing their use for etiopathological studies and drug screening tests based on blood–brain barrier (BBB) permeability, as well as a platform for studying neurological disorders and particularly cerebrovascular diseases [14, 15, 23–25]. Here, we aim to review both the current state and future perspectives of vascularized human brain organoids.

## Neural Organoid Generation

One of the first attempts to generate brain organoids that resemble the human brain in 3D was performed in 2013 by Lancaster and colleagues [13]. The original protocol relied on embedding PSCs in a basement membrane-like matrix to facilitate neuroepithelial development. The generation of brain organoids in this way primarily relied on intrinsic signals, thus requiring only a minimal number of growth factors and other substances for patterning, with basic fibroblast growth factor (bFGF) being used as the sole growth factor in the first 6 days. These self-developing organoids resulted in a stochastic mixture of different brain cellular components, spanning from the retina to the hindbrain, implying low reproducibility and high variability. To overcome this aspect, subsequent protocols were developed progressively introducing inductive cues such as morphogens and signaling molecules, capable of directing the neurodevelopmental specification in a timely manner [26, 27]. Indeed, by using these protocols, 3D structures with a more specific regional identity such as the hypothalamus, midbrain, brainstem, and choroid plexus could be created [28, 29]. Organoids’ growth and maturation can continue over several months, reaching a width of several millimeters [16]. Eventually, they contain a variety of different cell populations of ectodermal origin, including multiple neuronal subtypes, astrocytes, oligodendrocytes, and outer radial glia cells [27, 30–35]. Notably, it has been described that microglia can develop within the brain organoid, simultaneously with neuroectodermal cell types, if dual SMAD inhibition is removed. Indeed, innately developed mesodermal progenitors are capable of differentiating into mature microglia under the influence of the CNS microenvironment provided by neuroectodermal cells [36].

Human brain organoids have the advantage of being able to model unique human-specific characteristics, such as the development of outer radial glial cells which largely contribute to the development of the human cerebral cortex and whose alteration can be responsible for pathological conditions [15]. Different patterned CNS organoids’ derivation protocols have been implemented over the past few years. Telencephalic aggregates were first developed to segregate GABAergic and glutamatergic neurons, while cortical glutamatergic neurons and astrocytes have been derived through the generation of cortical spheroids [26, 37]. By modifying the neurocortical induction protocol, the choroid plexus and the medial pallium-like tissues, precursors of the hippocampal telencephalic area, were established [38]. Finally, midbrain organoids containing dopaminergic neurons of the nigro-striatal pathway were generated [39–41].

## The Neurovascular System

Under physiological conditions, the brain’s high metabolic demand is met by a network of capillaries that supply oxygen and nutrients. Since CNS organoids are derived from neuroectodermal tissue, they lack cerebral blood vessels which are of mesodermal origin. As a result, organoids lacking proper vascularization have suboptimal nutrient supply, resulting in reduced cell proliferation and loss of cell viability in their inner regions, premature differentiation, abnormal neurogenesis, and impairment of cortical development [13, 18–21].

Even though brain organoids can be maintained in culture *in vitro* for over a year, growth often ceases after a few months, with cells dying in the core. As a matter of fact, nutrients cannot reach cells located more than 200–400 µm from the surface [13]. Specific devices such as orbital shakers or bioreactors have been used to promote oxygen and nutrient supply, even if these strategies cannot replace the development of a proper neurovascular system [13, 42]. The establishment of an efficient neurovascular system within brain organoids is a crucial advancement in the field, as it also allows the replication of early developmental processes related to angiogenesis [14, 15, 23, 24].

In humans, brain vascularization occurs simultaneously with brain development. Vasculature defects may result in severe brain malformations at this stage, indicating how critical it is for the development of the human brain [43]. Brain blood vessels develop sequentially through accurate angiogenic processes in vertebrates, with new blood vessels stemming from pre-existing ones, as opposed to de novo vasculogenesis [44]. In the early stages of neuroepithelial development, endothelial cells (ECs) arise from mesodermal angioblasts and colonize the neuroepithelium via the perineural vascular plexus [45]. Subsequently, ECs coalesce into primitive vessels to become mature blood vessels [46].

**Fig. 1:**
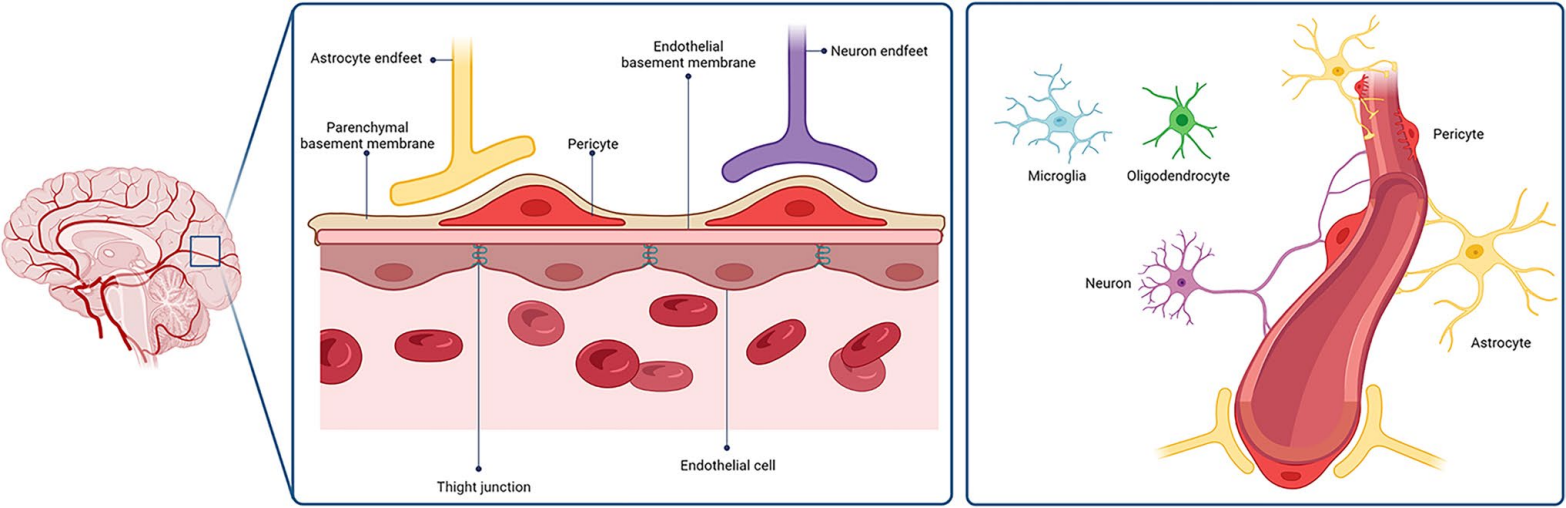
Representation of the neurovascular unit’s structure (NVU) and its constituent cell populations. Created in BioRender.com

The neurovascular unit (NVU) is formed by blood vessels interacting with neural and glial cells in the CNS, consisting of microvascular ECs, surrounding neurons, astrocytes, pericytes, and matrisomes [47] (Fig. 1). It regulates blood flow through a process called neurovascular coupling, provides trophic support, produces growth factors and paracrine signals, guides neuronal differentiation, and recruits oligodendroglia [48, 49].

The NVU can be modeled *in vitro* using different cell types derived from human PSCs which can be grown in monocultures or co-cultures to mirror interactions or can be cultured on microfluidic devices that mimic complex microenvironments [50]. These *in vitro* models can potentially be used to engineer artificial functional blood vessels [51]. Despite progress in the field, the generation of vascularized organoids using PSCs remains a challenging task which requests the precise coordinated supply of signals from multiple germ layers and tissue-specific microenvironmental cues involved in organogenesis [52].

Fully vascularized human brain organoids may allow researchers to study how NVUs’ develop and function in humans. Indeed, the pathogenesis of neurological disorders, strokes, and tumors has been linked to neurovascular dysfunction [53, 54]. As regards neurodegenerative diseases, an altered vascular system in the brain has been linked to increased BBB permeability, neurovascular uncoupling, oxidative stress, inflammation, and dysregulation of autophagy, all contributing to the development and maintenance of neurodegenerative processes [55–57]. For instance, in Alzheimer’s disease (AD) and vascular dementia, vascular damage and the breakdown of the BBB are associated with microcirculation damage and transcriptional changes in cerebrovascular cell types [58].

## Methods for Generating Vascularized Human Brain Organoids

**Fig. 2:**
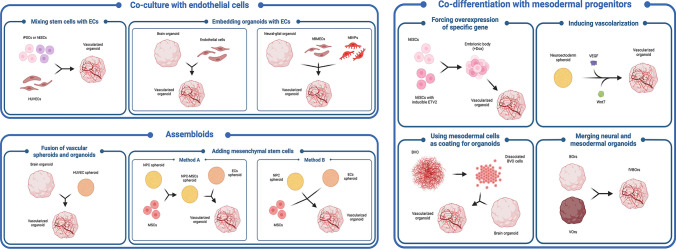
Schematic pictorial of different *in vitro* strategies for the generation of vascularized brain organoids. hESCs, human embryonic stem cells; iPSCs, induced pluripotent stem cells; hBMECs, human brain microvascular endothelial cells; hBVPs, human brain vascular pericytes; HUVECs, human umbilical vein endothelial cells; MSCs, mesenchymal stem cells; NPCs, neural progenitor cells; BVOs, blood vessel organoids; BOrs, brain organoids; VOrs, vessel organoids. Created in BioRender.com

**Fig. 3:**
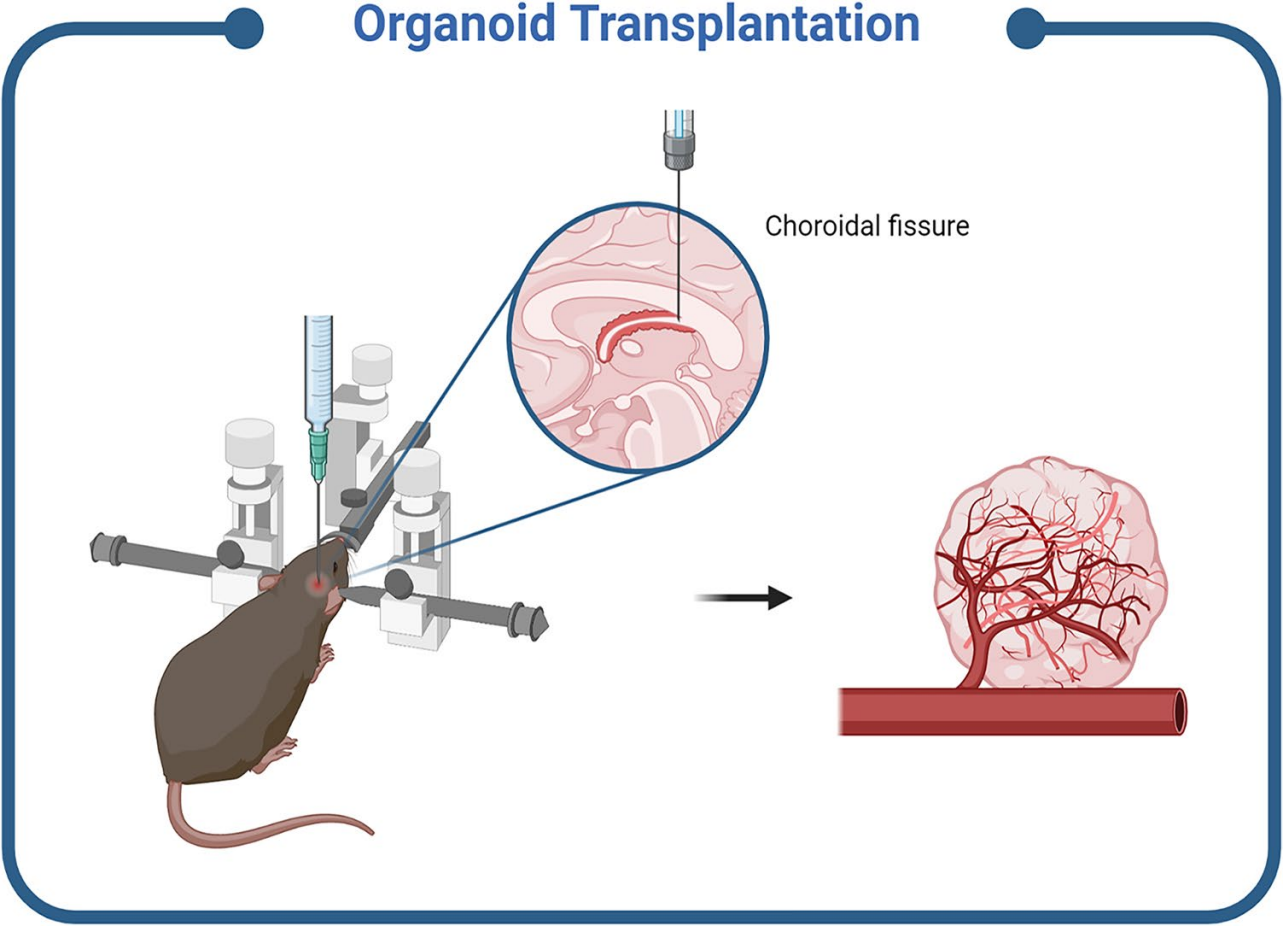
Schematic representation of *in vivo* strategy for the generation of vascularized brain organoids. It is important to notice that the type of injection reported in the pictorial is an example of a region-specific targeted *in vivo* transplantation approach [73]. Created in BioRender.com

Different strategies have been employed to induce vascularization within brain organoids. Some approaches involved the assembly of brain organoids with mesodermal progenitors or vascular organoids or using microfluidic devices to mimic vessels (Fig. 2). In contrast, other approaches included *in vivo* steps, such as transplanting organoids into immunodeficient mice and allowing host blood vessels to migrate into the grafts (Fig. 3). Vascularization strategies have multiple goals. Primarily, these techniques should allow the development of a branched vascular bed capable of adjusting changes in oxygen consumption, tissue growth, and metabolic needs of the organoid. Furthermore, organoid vascularization helps to preserve the cytoarchitecture, as well as favors the formation of a functional neurovascular unit and of the BBB. The achievement of these criteria would be an ambitious task, but a combination, even partial, of these elements, would ensure a faithful reproduction of CNS circulation. Currently, most *in vitro* models that have been developed lack an active blood flow, despite the presence of branching vessels. Excluding the vascularized organoids transplanted *in vivo*, the majority have not been demonstrated to have perfusable vessels. Therefore, benefits of perfusion and permeability could not be assessed, as well as biological processes in which blood flow or vascular tone is important, even though they reproduce early developmental processes such as angiogenesis and model trophic interactions between the BBB and the neurons. A notable exception is the work of Salmon et al. where vascularization and active perfusion of an iPSC-derived organoid is achieved with the aid of a custom designed 3D printed microfluidic chip [59]. Microfluidic devices and organ-on-chip systems may be a promising approach to perfuse neurovascular organoids, as described below.

### In Vitro Establishment of Vascularized Human Brain Organoids

As of today, many groups have developed methods for generating vascularized brain organoids *in vitro* (Table 1). A significant obstacle to the creation of vascularized brain organoids is the difficulty of coordinating induction factors for different germ layers and cell fates, as they often inhibit each other, such as ECs of the mesodermal origin and neural cells of ectodermal origin. As a result, many approaches have relied on separate cultures for the development of brain and vascular organoids [60].

**Table 1:** *In vitro* experimental strategies to vascularize human brain organoids

Starting cells for brain organoids	Mixed cells to model brain vessels	Organoid maturation level at combining	Method	Results	Ref
hiPSCs	hiPSC-derived ECs	DIV34	Embedding of 1 BOr and 250.000 ECs in one Matrigel drop. Co-culture for 3–5 weeks	Formation of human CD31 + penetrating vessels	[63]
15% HA 5% HM 15% HO 20% HN	30% HBMEC 15% HBVP	DIV2	Coating the surface of the neuroglial organoid by adding HBMEC and HBVP	• Coverage of the neuroglial organoid with CD31 + and PDGFRB + cells at DIV21 • Tight and adherens junctions are expressed	[66]
hESCs	hESC expressing ETV2 under *dox* promoter	DIV40-50	Organoid generation with at least 20% ETV2-expressing hESCs. Doxycycline-mediated ETV2 induction at DIV2 and DIV18	• Formation of a vasculature-like structure integrated in the organoid network, surrounded by astrocytes and pericytes • Vascularized organoids showed: - Increased growth - Reduced apoptosis - Accelerated maturation - BBB-like features - Enhanced expression of tight junctions and nutrient transporters - Higher trans-endothelial electrical resistance (TEER)	[53]
hiPSCs to obtain cortical NPC spheroids	iPSC-derived EC spheroids and human MSCs	DIV0-DIV7-DIV17 according to method A or B	**Method A**: MSCs were added to DIV7 NPC spheroids or directly combined at DIV0. At DIV14, EC spheroids were moved into the wells with DIV14 NPC-MSC spheroids **Method B**: EC and NPC spheroids were mixed at DIV14 of maturation, following by the addition of MSCs	• Formation of vessel-like structures interacting with neuronal population • Increased BBB gene expression (e.g., GLUT-1), CD31, and tight junction protein ZO1 expression	[68]
hESCs or hiPSCs	HUVECs	DIV0	Co-plating of hESCs or hiPSCs and HUVECs and co-culturing for 200 days to generate organoids	• Effective vascularization starting from the VZ-like zone • Bigger size of vascularized organoids • Decreased number of HIF1α + cells and cleaved Casp3 + cells • Expression of P-glycoprotein in HUVECs • Increased spontaneous firing at electrophysiological analysis in vascularized organoids	[61]
hESCs	N/A	N/A	Administration of VEGF (50 ng/mL) from DIV1 to DIV10, and then after Matrigel embedding of neuroectoderm spheroids, concentrations was reduced to 25 ng/mL VEGF. At DIV60, addition of Wnt7a to medium at concentration 10 ng/mL	• Vascularization induced by VEGF does not inhibit neural differentiation of EBs • Formation of CD31 + blood vessels in BOrs • Long-term BOrs culture with VEGF and Wnt7a results in vascular tubes with a two-layer structure • Increased expression of genes associated with vascular development, neurogenesis, and BBB	[70]
hiPSCs	hiPSC-derived mesodermal cells and angiogenic ECs	DIV15 BVOs into DIV4 BOrs	• Generation of hBVOs from hiPSCs until DIV15. Angiogenic ECs assembled at DIV5 of growth • hBVOs were dissociated at DIV15 and co-cultured with DIV4 BOrs	• Generation of CD31 + endothelial networks surrounded by SMA + SMCs and PDGFRβ + PCs • hBVOs showed a higher density of PCs than ECs, compared with the normal conditions of blood vessels in the brain (ratio 1:1) • Co-culturing of hBVOs and hCOs (Cerebral Organoids) developed vhCOs • Detection of collagen IV + basement membranes, CD144 + or KDR + endothelial progenitor-like cells and TUJ1 + neuronal cells into vhCOs • Detection of tight junction (ZO-I) and astrocyte (GFAP) markers	[71]
hESCs	hESCs (mesodermal induction and endothelial differentiation)	DIV0 (co-culture)	• Generation of VOrs from hESCs • Inclusion of brain EBs and VPs in one Matrigel drop • Co-culture till DIV40	• VOrs gradually surrounded BOrs generating fVBOrs at DIV40 • Detected CD31 + ECs, PDGFRβ + PCs, SMA + SMCs in VOrs • No CD31 + ECs were detected in BOrs • Identified several cell clusters in VOrs alone • fVBOrs showed significant increase in the thickness of neuroepithelial rosettes compared to BOrs (at DIV25) • BOrs at DIV40 revealed the presence of apoptotic Casp3 + cells in the central regions, whereas fVBOrs showed far fewer apoptotic cells	[72]
hiPSCs	HUVEC/HDF/hUCB-MSCs spheroid ratio (1:4:5)	DIV13 BOrs with DIV10 spheroids	• Generation of cerebral BOrs from hiPSCs • Formation of vascular spheroids from aggregation of HUVEC, HDF, and hUCB-MSCs • Fusion of vascular spheroids with BOrs	• Induction of angiogenesis in BOrs (CD31 + /vWF + /CDH5 +) • Promote cell proliferation and neurogenesis (Ki67 + /NeuN +) • Decreased apoptosis (lower levels of TUNEL + /rH2AX + /cleaved Casp3 + cells) • Upregulated Wnt/β-catenin signaling supports proliferation and neurogenesis observed in vascular organoids	[69]

*hiPSCs*, human-induced pluripotent stem cells; *ECs*, endothelial cells; *DIV*, days in vitro; *BOrs*, brain organoids; *HA*, human astrocytes; *HM*, human microglia; *HO*, human oligodendrocytes; *HN*, human neurons; *HBMEC*, human brain microvascular endothelial cells; *HBVP*, human pericytes; *hESCs*, human embryonic stem cells; *NPC*, neural progenitor cells; *MSCs*, mesenchymal stem cells; *BBB*, brain–blood barrier; *HUVECs*, human umbilical vein endothelial cells; *BVOs*, blood vessel organoids; *SMCs*, smooth muscle cells; *PCs*, pericytes; *hCOs*, cerebral organoids; *vhCOs*, vascularized hCOs; *VOrs*, vessel organoids; *EBs*, embryoid bodies; *VPs*, vascular progenitors; *fVBOrs*, fused vasculature and brain organoids; *HDFs*, human dermal fibroblasts; *hUCB-MSCs*, human umbilical cord blood-derived mesenchymal stem cells

Multi-lineage assemblies can be created also by adding non-ectodermal cells or their progenitors directly to brain organoids. A number of these approaches have been used, including the use of umbilical endothelial cells, mesodermal progenitors, and stem cells expressing a transcription factor for vascular lineage specification. With these experiments, organoids with tube-like vascular structures were generated [53, 61, 62]. Although, depending on the applied protocol, those multi-lineage approaches were variably able to originate perfusable vessels, they provided a model of human neurovascular interactions during development.

Pioneering work with multi-lineage assembly was from Pham and colleagues, which represents one of the first attempts to generate syngeneic structures. They generated both brain organoids and ECs from the same patient-derived iPSCs and then co-cultured them after differentiation [63]. Mesodermal cells were inducted with Wnt activation (e.g., CHIR99021) and differentiated into endothelial progenitors using BMP4, VEGF, and FGF2. On day 34, brain organoids were re-embedded in polymerized Matrigel droplets with endothelial progenitors and grown *in vitro* for 3–5 weeks, leading to robust vascularization. Hence, although the cytoarchitecture of the neuronal compartment was not very carefully described, organoids showed several penetrating and likely perfusable vessels expressing the human endothelial marker CD31, showing that patient-derived ECs can be used for brain organoids vascularization [63].

A couple of years later, Shi and colleagues used a similar co-culture strategy, but they grew human brain organoids with human umbilical vein endothelial cells (HUVECs) [61]. Of note, vascularization occurred first in the ventricular zone (VZ)-like region of the brain organoid populated by neural progenitor cells (NPCs), thus replicating the development of the human brain vasculature. HUVEC-vascularized human brain organoids displayed a reduced number of cells expressing the hypoxia marker HIF1α (hypoxia inducible factor 1 subunit alpha) and of cells undergoing apoptosis as indicated by caspase3 positivity, suggesting that vascularization is able to preserve cell survival. Furthermore, the authors observed in HUVECs the expression of different proteins involved in the development of vasculature compared to HUVECs grown in monocultures. As a matter of fact, HUVECs embedded in brain organoids expressed P-glycoprotein, suggesting that neural cells can influence gene expression and NVU-forming EC commitment. Further, according to single-cell RNA sequencing analysis of human brain organoids, vascularization appears to accelerate neurogenesis. Indeed, vascularized brain organoids, beside a cortical spatial cytoarchitecture that quite well recapitulated the cerebral compartment, contained more spontaneously firing neurons compared to non-vascularized ones [61].

A different strategy was tested by Cakir and colleagues, which exploited a genetic modification to induce EC differentiation during brain organoid formation [53]. They generated human cortical organoids from human ESCs, engineered to ectopically express human ETS variant 2 (ETV2). They used lentivirus-encoded ETV2 under doxycycline (dox)-inducible promoter to transduce hESCs. The activation of ETV2 started during neuronal induction at day 2 with low levels of doxycycline and was fully completed during cortical differentiation at day 18. Cells expressing ETV2 formed a vasculature-like structure within the cortical organoids, supporting increased growth, reduced apoptosis levels, and accelerated maturation. Indeed, the neural cytoarchitecture was improved and ETV2-induced EC showed specific BBB-like characteristics, including increased expression of specific proteins and nutrient transporters and trans-endothelial electrical resistance (TEER), which likely contributed to the functional maturation of neurons. An important feature of this model was that vessel-like structures were surrounded by astrocytes and pericytes, closely mimicking the composition of human NVU. Overall, a single inducible transcription factor-mediated approach can overcome the problems associated with the need for different culture conditions to induce vascularization as well as to control the precise timing of cell fate induction in intact organoids. Additionally, this approach may be used to refine the NVU model by generating proper BBB cell types [53]. The vascular system proved perfusable capacity upon *in vivo* grafting in mice.

Of note, in the last decade, the complex cell type arrangement of the BBB has been deeply investigated, showing that spontaneously ECs, pericytes, and astrocytes self-organize in a multicellular structure suggesting that the formation and cellular architecture of the BBB are inherently encoded within individual cell types [64, 65].

With a different approach, the team led by Atala generated neural units starting from different cell types such as human astrocytes, microglia, oligodendrocytes, and neurons [66, 67]. Neuroglial compartments were allowed to form for 48 h, before the addition of human brain microvascular endothelial cells (HBMEC) and human pericytes (HBVP). Tight and adherens junctions as well as BBB transport proteins were rapidly expressed in these vascularized cellular gatherings. By using this model, the authors were able to assess the neurotoxicity of molecules that cross or disrupt the BBB, albeit the neural cytoarchitecture was not characterized [67].

As an alternative approach, Song and colleagues proposed the combination of neural progenitor spheroids with EC spheroids and iPSC-derived mesenchymal stem cells both responsible of brain organoid vascularization [68]. They generated hybrid NVU assembloids with MAP2 + neurons, GFAP + astrocytes, and CD31 + ECs. These structures expressed typical BBB markers including GLUT1 and efflux transporter breast cancer-resistant protein (BRCP), tight junction markers like zonula occludens-1 (ZO-1), the matrix metalloproteinase remodeling proteins (MMP)-2 and MMP-3, collagen IV, laminin, and chondroitin sulfate proteoglycan as well as other matrix metalloproteinase remodeling proteins [68]. The spheroid fusion strategy offers several advantages compared to the direct mixing method, because it eliminates the need for cell dissociation, it allows the creation of hybrid spheroid structures with carefully arranged compartments, giving researchers greater control over the process. Moreover, this controlled arrangement facilitates the secretion of VEGF-A through the assembly of cortical spheroids, vascular spheroids, and mesenchymal cells, thereby accelerating cortical tissue development with hint of layer distribution.

Similarly, Kang’s group generated vascularized organoids by fusing iPSC-derived brain organoids, with human dermal fibroblasts, human umbilical vein endothelial cells, and human umbilical cord blood-derived mesenchymal stem cells, through the use of a non-adherent microwell culture system. Immunostaining analysis demonstrated the presence of well-organized vascular structures, reduced apoptosis, and increased Wnt/β-catenin signaling, albeit perfusion was not properly ascertained [69].

Ham and colleagues used a different approach in which vascular differentiation cues were added early on to immature neuroectodermal spheroids [70]. During EB formation and neural induction stages, recombinant human vascular endothelial growth factor (VEGF) was added. It was observed that vascularization was enhanced, without a significant reduction in neuronal markers. After 2 months, Wnt7a was added in the presence of VEGF in order to induce maturation and penetration of blood vessels, leading to capillary embedment in smooth muscle actin-positive pericytes [70]. Albeit both neuronal and, primarily, vascular structures have been characterized and neuronal structure benefitted from vascularization, perfusion capacity was not properly tested.

Since during human development vascularization begins outside of the CNS, Ahn and colleagues attempted to generate blood vessels and to promote sprouting of differentiated vessels within cortical organoids, rather than to induce typical vasculogenesis within the brain organoid [71]. Actually, after hBVOs were generated, they were dissociated into single cells to be co-cultured with cortical organoids and to create vessels that simulate embryonic neurovascular development. After 13 days of co-culture, CD31 + vessel-like structures were detected on organoid surfaces. Although the authors do not provide a specific characterization of the neuronal cytoarchitecture, beside tubulin beta-III positive cells, the expression of multiple BBB-specific markers indicated that hBVOs provided neural organoid vascularization [71].

A similar approach was recently tested by Sun and colleagues, who generated neurovascular assembloids by fusing brain and vascular organoids [72]. hBVOs were obtained upon mesodermal fate induction in H9 hESCs, followed by endothelial differentiation. Meanwhile, brain organoids were also differentiated from the same hESCs. The fusion process was initiated by positioning two young GFP-labeled hBVOs at opposite ends of one neuroectodermal body. After a few days, GFP-tagged cells coated and invaded the brain organoid. By day 40, the neurovascular assembloid with a vascular system embedded within the brain-like tissue was observed. The vascular system was able to positively promote neural development based on the number of neural progenitors present in the vascularized structures. No major consequences have been reported on the immunostained cortical layers that were described. Of note, assembloids were colonized by BBB-like structures and some microglial cells, which were responsive to stimuli and were able to phagocyte synapses [72]. Nicely, microcapillary testing proved perfusion properties.

Despite the presence of branching vessels in many of these models and of proved or predicted perfusion capacity, the main limitation in all of them is the absence of active blood flow, albeit blood cells are of mesodermal origin as well. Another limitation is connected with the fact that vascular development within the CNS occurs via an angiogenic process, rather than via primary vasculogenesis; therefore, models which consist in the fusion of pre-existing vessels with brain organoids are probably recapitulating more faithfully the physiological events occurring in human development [70, 72]. Furthermore, in some of these studies, *in vitro* cultures were engrafted *in vivo* to test organoid perfusion [53, 61, 63]. Alternatively, microfluidic culture devices may be a promising *in vitro* approach to test those properties. All in all, in spite of these limitations, brain organoids can be vascularized, albeit without blood flow, to reproduce early developmental processes such as angiogenesis and vascular structures can promote neural development and BBB formation in human brain organoids.

### Transplantation of Human Brain Organoids to Establish Circulation In Vivo

One of the first approaches to generate vascularized cerebral organoids was via their engraftment in animal models (Table 2). A xenotransplant into a vascular-rich niche exploits the natural angiogenesis of the host blood vessels, which colonize the engrafted cerebral organoids [73]. To achieve this goal, brain organoids were transplanted into adult immunodeficient mice’s cortex, where they are then invaded by host blood vessels and actively nourished. Organoid grafts were able to survive for a long period of time, to lower cell death and to enhance their own functionality and survival. Similar approaches have been employed to vascularize other immature organoid systems such as the liver, gut, kidney, and lung organoids [74, 75].

**Table 2:** *In vivo* strategies for transplantation of vascularized human brain organoids

Mouse model	Site of transplantation	Vascularized brain organoid	Organoid maturation day at transplantation	Results	Ref
NOD-SCID	Retrosplenial cortex (Brain)	hESC-derived human cerebral organoids	DIV40-50	The engrafted organoids generated a system of vessels perfused with host blood	[73]
Immunodeficient mouse	Brain	hiPSC-derived endothelial cells (ECs) co-cultured with whole-brain organoids	DIV54	Vascularization was found in the core of the organoids after transplantation, while *in vitro* remains external	[63]
Rag2^−/−^/GammaC^−/−^ immunodeficient mouse	Subcutaneous injection in the hind limbs	Organoid generation with at least 20% ETV2-expressing hiPSCs	DIV40-50	Pre-vascularization of the organoids extended their survival up to 30 days after transplantation	[53]
NOD-SCID	S1 cortex	Human brain organoids co-cultured with HUVECs	DIV60	Pre-vascularized organoids enhanced vasculature formation and attenuated apoptosis	[61]
NOD-SCID	Retrosplenial cortex (Brain)	hiPSC-derived human brain organoids transplanted with a graphene microelectrode array	DIV60	Functional integration between the organoid and the host brain	[76]

In detail, Mansour and colleagues developed an *in vivo* functional vascularized model by engrafting hESC-derived brain organoids in the brain of immune-deficient non-obese diabetic/severe combined immunodeficiency (NOD-SCID) mice [73]. After implantation into the retrosplenial cortex, grafts survived for several months. During this time, there was a gradual differentiation and maturation of neurons with axons sprouting into host tissue, synaptogenesis, and gliogenesis, including microglial invasion. Interestingly, murine vessels started to migrate toward the donor graft 1 week after implantation. A retro-orbital injection of dextran dye confirmed the existence of a perfused vascular network. Among engrafted organoids, 85.4% were vascularized, whereas non-vascularized did not survive, suggesting that blood flow supported their survival by delivering oxygen and nutrients. In addition, the engrafted ones had a larger size, a lower level of apoptosis, and more mature NeuN + neurons compared to those grown *in vitro*. Furthermore, *in vivo* imaging indicated the presence of functional neuronal networks and blood vessels, as well as synaptic neuronal activity between graft and host as assessed with extracellular recording combined with optogenetics [73]. Recently, Wilson and colleagues transplanted human cortical organoids into the retrosplenial cortex of adult mice and monitored their longitudinal function using a multimodal combination of transparent microelectrodes and imaging with two-photon confocal microscopy. These experiments confirmed the functional integration and the vascularization of the organoid by the host mouse brain [76]. Other groups transplanted brain organoids pre-vascularized through different co-culture methods with ECs or EC-like cells [53, 61, 63].

For instance, Pham and colleagues co-cultured whole-brain organoids with iPSC-derived ECs for 54 days and transplanted them into the brain of immunodeficient mice. *In vivo*, organoids survived up to 2 weeks, and vascularization was observed inside and between rosettes within the center, in contrast to non-transplanted organoids showing only external vascular structure [63].

Similarly, Shi and colleagues developed *in vitro* vascularized human brain organoids by co-culturing ESC- or iPSC-derived brain organoids with HUVECs for 2 months and then implanted them into the cortex of NOD-SCID mice. In transplanted brain organoids, both HUVEC-derived and mouse-derived ECs coexisted within capillaries, which demonstrated graft vessel integration into the host vascular system. The injection of fluorescent dextran dye confirmed structural integration [61].

Cakir and colleagues vascularized brain organoids through ETV2-transcription factor-mediated iPSC differentiation into ECs and then transplanted them into immunodeficient mice hind limbs. MRI images showed that pre-vascularized organoids survived for a month after transplantation, while non-vascularized control organoids disappeared between 10 and 30 days, being grafted in the hind limb and suggesting that *in vitro* generated vascular system is needed for connection with host blood flow network. A dye injection revealed infiltration and functional perfusion by the host vessels [53].

Overall, brain organoid engraftment *in vivo* overcomes the necrotic core limitation, providing perfusion and improving organoid viability. Although blood vessels in these models are partially murine, limiting their translatability to humans and restricting their use for high-throughput drug screening, all these studies support the significance of the engraftment of human brain organoids *in vivo* to create novel disease models.

## Development of Vascular Networks Through Microfluidic Devices

Over the past decade, many experiments have been conducted to recreate vascular structures *in vitro* using microfluidic devices [77]. Most vascular models were created *in vitro* using either vascular templating models, wherein a network of microfluidic channels is coated with ECs [78–80], or self-assembly, which prompts a microvessel network that self-organize lead to the establishment of a stable anastomosis with neighboring microfluidic channels [81, 82].

Recently, many studies have provided additional implementations on modeling blood vessel networks through synthetic devices [83–86]. Salmon and colleagues showed that cortical organoids (COs) can be vascularized through an avascular hydrogel matrix placed between two perfusing microfluidic channels [59]. In this environment, thanks to gradients of signaling cues, ECs can proliferate, move toward the organoids, and create a network of highly branched vessels. Albeit an increased expression of functional vascular markers, only in few COs’ vessels were able to penetrate their core. Nonetheless, an active perfusion of the neurovascular network was performed at day 20 and day 25, and while fluorescein-40-kDa dextran and 1-μm red fluorescent-beads underwent quick diffusion, bigger beads remained in the vessels, ascertaining vessel functional perfusion and permeability to small compounds [59].

Surely, organ-on-chip is an innovative technique that exploits multi-channel microfluidic systems to reproduce some biological and functional features of human parental tissues, such as the vascular system [87]. These devices are typically composed of transparent polymeric microchannels accessible to perfusing cells that will organize in 2D or 3D. This is a well-established technology for the intestine, bone marrow, liver, pancreas, heart, and brain organoids, which can be vascularized on chips [81, 86, 88–90].

Maoz and colleagues recently developed a linked organ-on-chip model of the NVU. This model was used to show metabolic coupling between neurons and vessels, with increased flux of neurotransmitters, especially the GABA one [91]. Being most of the common neurological diseases, including Parkinson’s disease (PD), AD, and Huntington’s disease (HD), linked to a dysfunction in the BBB [92], a vascularized brain model will certainly be a great advancement for studying and understanding the pathophysiological mechanisms of these diseases. Further, organ-on-chip could be extremely useful to analyze the NVU cells using calcium imaging and electrophysiology. One of the main advantages of organ-on-chip technology is that it allows the generation and maintenance of vascularized brain organoids that have human both neural and vascular structures, unlike the approach of transplanting human brain organoids into mice, which results in the incorporation of murine vessels. Furthermore, genome editing techniques, such as CRISPR-Cas9 technology, can be more easily implemented to study the biological pathways underlying vascular and BBB functions. The future use of on-chip devices will provide many advantages in the field of neurological medicine. Nevertheless, to fully utilize these artificial models in the biomedical field, further technological improvements are required.

## Future Perspectives and Applications

Here, we have presented a review on the approaches used to create vasculature in human brain organoids. Their relevance and their advantages over non-vascularized organoids have been cited and discussed. By creating a vascularized human brain organoid, researchers may overcome limitations due to interspecies differences such as the number and distribution of neuronal cells, their biochemical properties, BBB permeability, and angiogenic processes [93–96]. Moreover, iPSC-derived organoids are patient-specific, and thus, data production can be matched across patients with common genetic background in a forward-looking perspective. Indeed, vascularized organoids closely mimic the *in vivo* setting, and they may become one of the most promising approaches for drug discovery or personalized medicine [97].

Moreover, understanding the molecular mechanisms underlying various diseases, including cerebrovascular disorders, neurodegeneration but also chronic-acquired diseases such as diabetes and hypertension, could be improved using a more amenable human NVU model. Indeed, several mechanisms are altered at NVU level and associated with AD, vascular dementia, and diabetes-induced neurodegeneration [48, 92, 98]. Moreover, different components of the NVU have been associated with cognitive impairment, including pericytes [99, 100]. Excluding *in vivo* animal NVU models, current human *in vitro* NVU models have been and are very useful [101, 102]; nonetheless, as 2D models, they (i) lack the opportunity to mimic the blood flow and shear stress, key components of the vascular cytoarchitecture and (ii) are unable to form perivascular cell–brain endothelial cell interaction and to reproduce BBB complexity. As 3D models with chips or microfluidic system, they suffer from (i) membrane porosity that limits cell–cell interaction; (ii) use of rigid extracellular matrix that impairs perivascular cell organization and viability; (iii) incapability to reproduce capillary dimensions and to recapitulate hierarchical *in vivo* branching; (iv) need of very specialized equipment; and (v) no possibility to measure trans-endothelial electrical resistance (TEER) [103].

In addition, vascularized organoids may also be useful for studying the development and plasticity of human NVU. In the developing brain, NVU cells secrete several factors that drive neuronal differentiation, migration, and synaptic plasticity [104, 105]. Research on rodent models has demonstrated that ECs could attract recently differentiated neurons to migrate along vessels and reach areas affected by ischemia [106]. The influence of neurovascular interactions on developmental (angiogenesis, BBB formation, mural and vascular cell development, regulation of neuronal and glial development) and reparative events in humans remains unclear since there is no relevant human model or there are models with poor human translatability. Indeed, *in vitro* conventional cell culture models [107], microphysiological systems [108, 109], tissue-engineered 3D models, or bioprinting [110] do not properly fulfill conditions of the human NVU microenvironment. Recreating the intricate microenvironment and the cellular interactions within the neurovascular unit becomes pivotal in comprehending its functioning and, further, in devising CNS-targeted pharmaceuticals that can effectively penetrate the brain for experimental testing. The development of vascularized organoids could overcome this obstacle. Additionally, gene expression analysis, including single-cell profiles, may reveal new players in neurovascular mechanisms.

Further, organoids could also prove extremely useful for regenerative therapies. Indeed, a differentiated human brain organoid, containing appropriate cell populations, active neural circuits, and an adequate vasculature may facilitate their use as a cell source for transplantation strategy for CNS tissue regeneration [73, 111]. Finally, vascularized human brain organoids hold the potential for application in drug screening, discovery of new potential disease biomarkers, and advancement of cutting-edge diagnostics and therapies [63, 73]. As clinical avatar, patient-specific organoids can be used for diagnostic interventions, tested with therapeutic strategies, and personalized medicine could eventually be achieved in the next future [97].

Overall, it appears clear that brain organoids, especially vascularized organoids, could prove to be a fundamental resource for understanding and treating neurological diseases.

## Conclusions

Vascularized human brain organoids are still imperfect tools, and they need to be further optimized before they are able to precisely recapitulate the development, function, and pathology of the neurovascular system. Several studies demonstrated that vascularizing human brain organoids allowed for a better maturation and survival of neural cells. More specifically, better functional circuit and firing rate [61] and increased number of mature neurons [73] have been observed. Furthermore, some key aspects of the interactions between vascular cells and neural cells in health and pathology have been clarified [53, 69], including the regulation of the BBB maturation by neural cues [72].

Current research suggests that vascularized brain organoids provide better pathophysiological models compared to non-vascularized ones [61, 68]. Indeed, compared to other *in vitro* models, neurovascular organoids can better reproduce the cytoarchitecture of the brain, providing functional and synaptic connectivity data. Nevertheless, the generation of vascularized brain organoids requires more resources with consequent lower accessibility than non-vascularized organoids, hindering their applicability. For an extensive use of brain organoids for disease modeling and drug discovery, substantial advancements in automation and scaling up are needed.

The pathophysiology of brain diseases is influenced by blood flow, cellular composition, vascular cell-derived factors, and microglia. Recent advances in cell culture technology allowed the growth of brain organoids for long periods, paving the way to model late-onset diseases such as neurodegenerative diseases [17]. Indeed, long-term cultures of vascularized brain organoids may be very useful for modeling aging-associated diseases such as stroke or AD, while young organoids used to model fetal brain development may not accurately reproduce stroke-relevant phenotypes.

Since vascularized organoids can more easily integrate into the host tissues and may promote healing better than non-vascularized organoids, they may be an excellent source for cell transplantation in regenerative medicine approaches. Nonetheless, more studies are required to evaluate the chance to perfuse vascularized brain organoids without transplanting them *in vivo*. Indeed, a controlled perfusion method could assess the vessel permeability through a direct injection of blood cells into the organoid as well as reduce the characteristic necrotic core of organoids [112, 113]. Additionally, biomechanical properties of the brain tissue and its vascular system such as stiffness, viscoelasticity, and spatial organization influence physiological processes such as proliferation, migration, differentiation, and cell functions [114].

Overall, all these studies have emphasized the significance of vascularized organoids in faithfully recapitulating different aspects of the neurovascular microenvironment, paving the way for a deeper understanding of the molecular mechanisms underlying neurodegeneration in a forward-looking perspective of identifying new therapeutic strategies.

## Data Availability

Not applicable.
